# Observations on the Prevention of Severe Invasions of Scarlatina

**Published:** 1849-08

**Authors:** Thomas Carroll

**Affiliations:** Cincinnati


*Observations on the Prevention of Severe Invasions of Scarlatina.*—By Thomas Carroll, M. D., of Cincinnati.

*To the Ohio Medical Convention:*

Gentlemen: The subject upon which I propose addressing you is one that has for a long time engaged my attention. The conclusions at which I have arrived are founded not upon ideal theory, as I believe, but upon the observations of facts which have not been confined within a narrow period, nor limited to a single locality. I think I have found them calculated to ameliorate much of the suffering produced by a most severe malady, and not unfrequently to aid in averting the hand of death from the sufferers. I therefore submit them to your consideration, in the hope that some of your body may be induced to put them to the test in their practice.

I first observed scarlatina during the year 1829. Between that period and 1832 I often witnessed it in various neighborhoods; and in some of these it was attended with great mortality. I had the misfortune, in the year 1831, to lose, by this disease, two patients in one family, within a few days; and some time during 1832 I lost two more, with the same affection, in another family. A third victim in one of these families was reserved for the hands of a steamer. These disasters sunk deep into my mind, and produced a train of thoughts, which led to the following conclusions:

That the attack of scarlatina might possibly be mitigated in its tendency to destroy life by preparing for its reception, by a strict course of dieting, and by a proper attention to the condition of the alimentary canal. I theredetermined that when I should again be called to treat a case of this disease in a family, which consisted of more individuals who might take the malady, to advise those to be subjected to a rigid course of dieting. That animal food should be prohibited, with the exception of milk—and that a mild cathartic should be administered to each individual, and be again repeated within the ensuing four or five days. After experience taught me that, where children were laboring under diarrhea, it was of great importance to effect a cure of it, if possible, before the invasion of the scarlatina.

This course I have pursued most undeviatingly ever since 1832, and I have, I believe, in all cases been able to persuade families to adopt it. The result has been that, since the time named, I have on no single occasion lost more than one case of scarlatina in a family during the continuance of any epidemic. Indeed, I do not now recollect that I have at any time lost more than one case in a family since that period.

The foregoing has been the result in my private practice; but at the Cincinnati Orphan Asylum my experience has been still further extended. In this institution I have met with the disease but once in an epidemic form, but on two or three occasions a very few sporadic cases have appeared. It very generally pervaded the inmates of the house in 1846, during the latter part of February, through March, and part of April. There were but about thirty children susceptible of the disease; of these one died. When the first case made its appearance, I directed a strict abstinence from animal food, and in two or three days a cathartic of one grain of calomel and ten of jalap was given to each inmate over two years of age, and half that amount to those under that age. But one set of purgatives was given; but the dieting was persevered in throughout, and I think with the happiest results; for all the cases, with the single exception mentioned, terminated favorably. Indeed, the disease was mild in nearly all the cases.

I am aware that cases may occur in which it would be improper to altogether withdraw animal food; and also that in some cases severe purging should be avoided, as in diarrheal cases where debility and anemia exist. Here it would be necessary to restrain diarrhea and remove debility. Indeed, in cases coming under these denominations, I have tried the plans mentioned with the happiest effects. Yet what can be done with the unhealthy can be best effected before the development of the specific affection, for all know how uncertain is the safety of a patient laboring under scarlatina, who has had it superadded to some chronic malady.

I am fully aware that a long succession of favorable results may occur where but little is due to any mode of medication. This may take place from the fact, that the disease very often appears in a mild form, and recovery is very general under any mode of treatment. But it must not be forgotten that I have been in the habit of pursuing the foregoing plan of preventing severe invasions of scarlatina during a period, now approaching twenty years; and whether in public or private practice it has but on one occasion disappointed me. I have now resided in Cincinnati more than eight years, and during more than seven years of this time I have been physician to the Cincinnati Orphan Asylum; and have, notwithstanding, lost but four patients by this disease, including both my private and public practice. I do not know that my mode of treating this malady is better adapted to prevent mortality than that of my professional brethren, and therefore I attribute whatever of superior success I may have had to the preparation of the constitution, in the manner indicated, for the reception of the disease.—*Ibid.*

